# Association of Polymorphisms within HOX Transcript Antisense RNA (HOTAIR) with Type 2 Diabetes Mellitus and Laboratory Characteristics: A Preliminary Case-Control Study

**DOI:** 10.1155/2022/4327342

**Published:** 2022-03-22

**Authors:** Saman Sargazi, Mahdiyeh Ravanbakhsh, Milad Heidari Nia, Shekoufeh Mirinejad, Roghayeh Sheervalilou, Mahdi Majidpour, Hiva Danesh, Ramin Saravani

**Affiliations:** ^1^Cellular and Molecular Research Center, Research Institute of Cellular and Molecular Sciences in Infectious Diseases, Zahedan University of Medical Sciences, Zahedan 9816743463, Iran; ^2^Pharmacology Research Center, Zahedan University of Medical Sciences, Zahedan, Iran; ^3^Department of Clinical Biochemistry, School of Medicine, Zahedan University of Medical Sciences, Zahedan, Iran; ^4^Department of Clinical Biochemistry, School of Medicine, Hamadan University of Medical Sciences, Hamadan, Iran

## Abstract

Type 2 diabetes mellitus (T2DM) is a complex heterogeneous disease resulting from the environment and genetic interactions. Lately, genetic association studies have shown that polymorphisms in long noncoding RNAs (lncRNAs) are associated with T2DM susceptibility. This preliminary study is aimed at investigating if HOX transcript antisense RNA (*HOTAIR*) polymorphisms contribute to T2DM development. Five hundred clinically diagnosed T2DM cases and 500 healthy controls were recruited from the southeast Iranian population. Genomic DNA was isolated from nucleated blood cells and genotyped for *MspI* (C/T) (rs920778) and *AluI* (A/G) (rs4759314) polymorphisms using the PCR-RFLP technique. For genotyping rs12826786 C/T and rs1899663 G/T variants, ARMS-PCR method was applied. Our findings indicated that *HOTAIR* rs920778 C/T, rs12826786 C/T, and rs4759314 A/G polymorphisms have a significant positive association with T2DM, while a negative association was observed between rs1899663 G/T T2DM susceptibility. Significant associations were also observed between rs920778 C/T and HDL-C as well as s4759314 A/G and both FBS and LDL-C in T2DM patients. Haplotype analysis indicated that the CGCG, CTTG, TGTA, and TTTG haplotypes of rs920778/rs1899663/rs12826786/rs4759314 significantly enhanced T2DM risk by 1.47, 1.96, 2.81, and 4.80 folds, respectively. No strong linkage disequilibrium was found between the four *HOTAIR* SNPs. We firstly reported that *HOTAIR* rs1899663 G/T, rs12826786 C/T, rs4759314 A/G, and rs920778 C/T polymorphisms might influence T2DM susceptibility by modulating different signaling pathways and could be regarded as potential prognostic markers in T2DM patients.

## 1. Introduction

Type 2 diabetes mellitus (T2DM) is the most common endocrine disease known as adult-onset or non-insulin-dependent diabetes [1]. Both obesity and decreased activity are common causes of insulin resistance which can lead to the onset and progression of T2DM [2]. The main clinical manifestations in diabetic patients include polyuria, polydipsia, and unexplained weight loss [3]. As a highly heterogeneous disorder, T2DM is considered the leading cause of microvascular (i.e., nephropathy, retinopathy, and neuropathy) and macrovascular complications and confers an increased risk of cardiovascular diseases [3–5]. Many factors increase the risk of developing T2DM, such as obesity, body mass index (BMI) ≥ 24 kg/m^2^, waist circumference (≥78 cm for women and ≥86 cm for men), smoking, inactivity, diet (high red meat, low fiber, high fat), and most importantly, genetics [6–8]. Recently, the incidence of T2DM has been increased in the America and the Middle East countries [9]. The incidence of this endocrine disease ranges from 2.6 to 15.1% in the Asia-Pacific countries and 3.5 to 13.1% in the Iranian population aged thirty or more [10].

Genetics plays a crucial role in the etiology of diabetes [11]. Single-nucleotide polymorphisms (SNPs) located in intergenic and/or intragenic regions of several candidate genes have been associated with T2DM risk [12]. Generally, the mammalian genome is comprised of coding sequences (less than 2% of the total genome) and noncoding (more than 90% of the total genome) [13]. Noncoding sequences lack the capacity for protein synthesis and participate in a broad range of cellular functions. These functions mainly include the organization of protein synthesis (mediated by ribosomal RNA and tRNA), regulation of protein synthesis (mediated by microRNAs), and regulate gene expression at transcriptional levels, which is mediated by long nonencoding RNAs (lncRNAs) [14, 15].

lncRNAs are more than 200 nucleotides in length and play fundamental roles in diverse biological processes, such as epigenetic modification, translation, and transcription control [16]. Recently, lncRNAs were found to be associated with T2DM in different ethnicities [17]. Studies have shown that lncRNAs are involved in glucose homeostasis and, therefore, contribute to the pathogenesis of diabetes and its complications [18]. Some studies have shown high heterogeneity of lncRNA expression in patients with T2DM, while others reported upregulation [19, 20] or downregulation [21, 22] of these noncoding RNAs in T2DM cases.

HOTAIR (transcription of HOX antisense RNA) is a well-studied lncRNA located on human chromosome 12q13 [20]. As a carcinogenic lncRNA located in the HOXC region with 2158 nucleotides and 6 exons, *HOTAIR* is epigenetically regulated via histone methylation and silences its target genes, including *HOXD* [23]. It has been established that HOTAIR serves pivotal functions in controlling cell growth, apoptosis, invasion, metastasis, and movement of malignant cells [24]. In addition, HOTAIR enhances atherosclerosis and induces oxidative stress by targeting miR-330 in macrophages, a microRNA involved in regulating insulin resistance [25]. Furthermore, HOTAIR activates NF-*κ*B (nuclear factor kappa-light-chain-enhancer of activated B cells) and subsequently upregulates LPS-induced glucose transporter 1 (GLUT1). This results in increased glucose uptake in macrophages [26]. So far, a limited number of studies have been conducted on the association between *HOTAIR* and insulin resistance in T2DM patients [20, 27]. Existing studies mainly investigated the role of lncRNAs in the etiology of metabolic disorders and diabetic complications [28, 29].

It was previously hypothesized that HOTAIR plays a crucial role in the regulation of glucose metabolism [30]. Still, not a single report is published on the association of four *HOTAIR* SNPs (rs1899663 G/T, rs12826786 C/T, rs4759314 A/G, and rs920778 C/T) with T2DM development. Herein, we designed this case-control study to investigate such association in an Iranian population.

## 2. Materials and Methods

### 2.1. Subjects

This case-control study was performed on a total of 1000 subjects (500 T2DM patients and 500 healthy individuals) from November 2020 to April 2021. T2DM patients were a mixture of new cases, and patients with uncontrolled T2DM admitted to the Diabetic Centers of Bu-Ali and Ali Asghar Hospitals, Zahedan, Iran. Diagnosis of T2DM was according to the criteria of the World Health Organization (WHO) in 2019 [31]. The control group was randomly selected from healthy persons with fasting blood glucose (FBS) < 100 mg/mL and hemoglobin A1c (HbA1c) < 5.7% which were resided in the same geographic area as patients and had no history of diabetes, inflammatory disease, metabolic syndrome, and cancer, as well as cardiovascular, renal, or hepatic diseases. Subjects in both groups were matched in terms of age, gender, and body mass index (BMI). BMI was calculated as body weight divided by height squared (kg/m^2^).

### 2.2. Sample Collection and DNA Isolation

Two milliliters of whole blood was drawn from each participant and collected into ethylenediaminetetraacetic acid (EDTA) tubes to prevent blood clotting. Genomic DNA was isolated using QIAamp DNA Blood Mini Kits following the manufacturer's instructions. After 12-14 h fasting, 3 mL of whole blood was also collected into heparinized tubes for biochemical measurements. HbA1C, fasting blood sugar (FBS), high-density lipoprotein- (HDL-) cholesterol, low-density lipoprotein- (LDL-) cholesterol, triglyceride (TG), and total cholesterol (TC) were assessed using commercially available kits (Pars Azmun Co., Tehran, Iran).

### 2.3. SNP Selection and Genotyping

**Figure 1:**
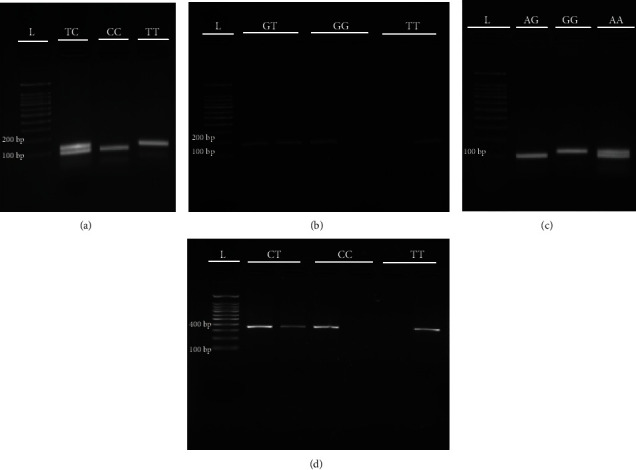
Genotyping of *HOTAIR* gene polymorphisms by polymerase chain reaction-restriction fragment length polymorphism (PCR-RFLP) ((a) rs920778 T/C, (b) rs4759314 A/G) and amplified refractory mutation system (ARMS-PCR) ((c) rs1899663 G/T, (d) rs12826786 C/T) methods resolved on a 2% agarose gel.

Four common HOTAIR gene SNPs (with minor allele frequencies higher than 0.095 based on information provided by 1000 genome projects) were selected based on their involvement in the susceptibility to various diseases [32, 33].  Table 1 presents the primers used for genotyping *HOTAIR* variants. Assessment of genotypic discrimination for three of the studied SNPs was done using polymerase chain reaction amplification-restriction fragment length polymorphism (PCR-RFLP) (for rs920778 C/T) and amplification refractory mutation system polymerase chain reaction (ARMS-PCR) (rs1899663 G/T and rs12826786 C/T) methods, as previously described [32]. Regarding rs4759314 A/G, a mismatch RFLP was established, and PCR conditions were 95°C for 6 min, 35 cycles of 95°C for 35 sec, 55°C for 35 sec, and 72°C for 35 seconds, followed by a final extension at 72°C for 5 min. PCR products were then electrophoresed on 1% agarose gel containing ethidium bromide (0.5 *μ*g/mL) and visualized using a Gel Doc imaging system (Figure 1). For quality control, at least 20% of the samples were randomly regenotyped, and results confirmed the 99% accuracy in genotyping.

### 2.4. Statistical Analysis

Deviation from the Hardy Weinberg equilibrium (HWE) was examined using *χ*2 goodness-of-fit test. Differences between sets of data were tested using the *χ*2 test and the independent sample *t*-test when appropriate. Adjusted odds ratios (ORs) with 95% confidence intervals (CIs) were calculated by multiple logistic regression analysis for the association between allele/genotype frequencies of *HOTAIR* SNPs and the risk of T2DM. All the analysis was performed using the SPSS (v.22) software. The SHEsis software was utilized to conduct haplotype analysis. A *p* < 0.05 was considered statistically significant.

## 3. Results

### 3.1. Clinical Features of the Study Population

The mean age was 54.87 ± 11.15 in controls and 55.28 ± 10.20 in T2DM cases. No significant difference was found among both groups in terms of age (*p* = 0.066) and sex (*p* = 0.290). The demographic and clinical characteristics of the studied population are shown in Table 2. Compared with controls, T2DM patients had markedly higher BMI, FBS, HbA1C, TG, and LDL-cholesterol levels (*p* < 0.001).

### 3.2. Genetic Association Analysis

None of the studied SNPs violated HWE in controls (*p* value for HWE > 0.05).  Table 3 demonstrates allelic and genotypic distribution of *HOTAIR polymorphisms*. All the *p* values were adjusted for age, sex, and BMI. Genotyping of rs920778 C/T showed that TT genotype significantly increased the risk of T2DM under codominant (OR = 1.93, 95% CI (1.27-2.92), *p* = 0.002), dominant (OR = 1.35, 95% CI (1.05-1.74), *p* = 0.021), and recessive (OR = 1.72, 95% CI (1.17-2.53), *p* = 0.006) inheritance models. As for rs12826786 C/T, enhanced risk of T2DM was observed under codominant CT (OR = 1.79, 95% CI (1.21-2.62), *p* = 0.003), dominant CC + CT vs. TT (OR = 1.36, 95% CI (1.05-1.76), *p* = 0.020), and recessive TT vs. CC + CT (OR = 1.57, 95% CI (1.10-2.22), and *p* = 0.010) contrasted genetic patterns. Likewise, codominant GG (OR = 1.61, 95% CI (1.07-2.42), *p* = 0.021) and recessive GG vs. AG + AA (OR = 1.52, 95% CI (1.04-2.23), *p* = 0.030) patterns of rs4759314 A/G conferred an increased risk of T2DM. Interestingly, codominant TT (OR = 0.51, 95% CI (0.33-0.80), *p* = 0.003) and recessive TT vs. CT + GG (OR = 0.53, 95% CI (0.35-0.81), *p* = 0.003) models of rs1899663 G/T were associated with protection against T2DM in our population. The T allele of rs920778 C/T and rs12826786 C/T and the G allele of rs4759314 A/G enhanced T2DM risk by 1.32, 1.30, and 1.21 folds, respectively. In contrast, rs1899663T allele decreased the disease risk by 19% (OR = 0.81, 95% CI (0.67-0.97), *p* = 0.022).

As shown in Table 4, we found significant associations between rs920778 C/T and HDL-C in T2DM cases (*p* = 0.016), rs12826786 C/T and HDL-C in controls (*p* < 0.001), rs4759314 A/G and FBS (*p* = 0.008) and LDL-C (*p* = 0.043) in T2DM cases, and rs4759314 A/G and TC (*p* = 0.041) and TG (*p* = 0.004) in controls.

### 3.3. Haplotype and Linkage Analysis

**Figure 2:**
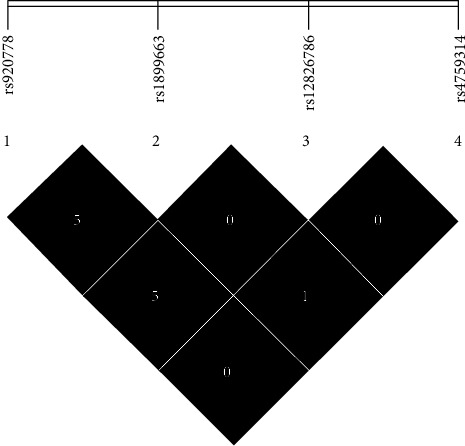
LD analysis between *HOTAIR* rs920778 C/T, rs1899663 G/T, rs12826786 C/T, and rs4759314 A/G SNPs. No strong linkage disequilibrium was found between the studied variations.

The analysis of haplotypes revealed that the CGCA haplotype of rs920778/rs1899663/rs12826786/rs4759314 was more frequent in both T2DM cases and controls (Table 5). We found that the CGCG, CTTG, TGTA, and TTTG haplotypes of rs920778/rs1899663/rs12826786/rs4759314 significantly enhanced T2DM risk by 1.47, 1.96, 2.81, and 4.80 folds, respectively. On the other hand, the CTCA haplotype of rs920778/rs1899663/rs12826786/rs4759314 diminished risk of T2DM in our population by 54% (OR = 0.46, 95% CI (0.31-0.69), *p* < 0.001). We also calculated the amount of linkage disequilibrium (LD) between four *HOTAIR* SNPs in the control group (Table 6 and Figure 2). The highest amount of linkage was found between rs12826786 and rs920778, which was about 3.2%. However, other LD values were below 2%, indicating no strong linkage between the studied variants.

## 4. Discussion

In the current study, for the first time, we aimed to assess the link between four noncoding *HOTAIR* polymorphisms and the risk of T2DM. All the studied variations have resided within intergenic regions of the *HOTAIR* gene. This is important since functional intronic variations can impact alternative gene splicing and the expression of remote genes at a distance [34]. We found an increased risk of T2DM under allelic, codominant homozygous, and recessive models of rs920778 C/T and rs4759314 A/G polymorphisms along with allelic, codominant homozygous, dominant, and recessive genetic patterns of rs12826786 C/T polymorphism. At the same time, codominant TT vs. GG, allelic T vs. G, and recessive TT vs. GT + GG models of rs1899663 G/T conferred protection against the risk of T2DM. Significant associations were also noticed between rs920778 C/T and HDL-C along with s4759314 A/G and FBS and LDL-C in T2DM cases. We conducted haplotype analysis and found a positive correlation between CGCG, CTTG, TGTA, and TTTG haplotypes of rs920778/rs1899663/rs12826786/rs4759314 and T2DM risk, while no strong LD was observed between the studied variants.

As a well-studied lncRNA, HOTAIR has been associated with several malignancies, such as gliomas, thyroid, liver, lung, breast, and colorectal cancers [35]. For example, Sathishkumar et al. reported the elevated levels of HOTAIR, metastasis-associated lung adenocarcinoma transcript 1 (MALAT1), myocardial infarction associated transcript (MIAT), X-inactive-specific transcript (Xist), antisense noncoding RNA in the INK4 locus (ANRIL), P21-associated ncRNA DNA damage-activated (PANDA), growth arrest-specific transcript 5 (GAS5), and neighbor of BRCA1 gene 2 (NBR2) in peripheral blood mononuclear cells of patients with T2DM, as compared with controls [36]. In contrast, Akerman et al. showed that PLUTO, a *β* cell-specific lncRNA, is downregulated in T2DM patients [37]. Another study showed that *HOTAIR* is overexpressed in liver tissues of T2DM patients [20].

Chen et al. showed that HOTAIR downregulates miR-17-3p in human articular chondrocyte cells [38]. Yet, there are conflicting results concerning miR-17 expression in T2DM cases. Chen et al. proposed that plasma miR-17 is upregulated in T2DM patients [39], while Karolina et al. found this microRNA to be downregulated in these cases [40]. In another study, Ma et al. suggested that HOTAIR targets miR-143 and regulates its expression [41]. We previously showed that miR-143 is associated with the risk of T2DM in a sample of the Iranian population [42].

On the other hand, it has been established that upregulated *HOTAIR* noticeably enhances hepatic insulin resistance by activating of Akt (protein kinase B)/glycogen synthase kinase-3 (GSK) signaling pathway [20]. Besides, HOTAIR upregulates a number of genes associated with cell cycle, including checkpoint kinase 1 (CHEK1), cyclin A2 (CCNA2), cyclin B2 (CCNB1), serine/threonine-protein kinase (PLK4), active-state power management (ASPM), and non-SMC condensin I complex subunit G (NCAPG), which was previously shown to be linked to T2DM development [43]. Qi and Zhong investigated the role of HOTAIR in the onset of diabetic cardiomyopathy (DCM). They suggested that this lncRNA increases the viability of cardiomyocytes by activating the phosphatidylinositol 3-kinase (PI3K/Akt) pathway [44]. In a similar study, Gao and colleagues reported that HOTAIR serves as a molecular sponge of miR-34a in cardiomyocytes, and sirtuin 1 was considered a target of miR-34a [45]. Therefore, HOTAIR overexpression could be associated with protection against DCM. On the contrary, Majumder and colleagues showed that dysregulated HOTAIR acts as a bystander in diabetic kidney disease without participating in the pathogenesis of this type of kidney abnormality [46]. Furthermore, as an active recruiter of chromatin-modifying complexes, HOTAIR mediates angiogenesis in diabetic retinopathy [28]. In silico analyses predicted that HOTAIR is involved in various diabetes mellitus-related pathways, including apoptotic cell death, tumor necrosis factor (TNF), ras-mitogen-activated protein kinase (MAPK), forkhead box O (FoxO), and hypoxia-inducible factor 1 (HIF1) [47]. This makes HOTAIR an important biomarker for diabetic chronic complications. Still, the precise role of HOTAIR in the etiology of T2DM has remained unknown.

Genetic variants in lncRNAs have been correlated with the risk of T2DM in Iranians [48]. Previously, studies have shown that *HOTAIR* polymorphisms remarkably enhance the risk of different types of cancers [49], preeclampsia [50], primary ovarian insufficiency [51], coronary artery disease [52], etc. In contrast, Bayram et al. reported that *HOTAIR* rs920778 C/T polymorphisms were not associated with gastric cancer risk in a Turkish population [53]. Wang et al. revealed that *HOTAIR* rs920778 C/T and rs1899663 G/T polymorphisms were linked to lung cancer susceptibility, smoking status, and gender of a Chinese population [54]. Based on the findings of Li et al., *HOTAIR* rs4759314 A/G influences the transcription efficacy of *HOTAIR* gene promoter. This results in dysregulation of HOTAIR/miR-545/epidermal growth factor receptor (EGFR)/MAPK pathway and, thus, is implicated in the pathogenesis of congenital heart disease [33]. In our study, we found a positive association between *HOTAIR* rs920778 C/T, rs12826786 C/T, and rs4759314 A/G polymorphisms and T2DM, whereas a negative association was observed between rs1899663 G/T and T2DM susceptibility.

It has been established that HOTAIR is involved in hepatic insulin resistance via inhibiting sirtuin 1 (SIRT1), a potential therapeutic target to combat insulin resistance and diabetes, and suppressing the/Akt/glycogen synthase kinase-3*β* (GSK-3*β*) pathway [55, 56]. Moreover, HOTAIR expression was found to be markedly increased following tumor necrosis factor-alpha gene (TNFA) stimulation [56]. TNFA contributes to the pathogenesis of a variety of inflammatory diseases as well as type 1 diabetes mellitus (T1DM) in children and adolescents [57, 58]. Irregularity in GSK-3*β* gives rise to diabetic encephalopathy [59], diabetic cardiomyopathy [60], maternal type 1 diabetes [61], T1DM [62], and other types of diabetes [63]. Jiang et al. proposed that *HOTAIR* downregulation is associated with decreased expression of insulin-like growth factor-1 (IGF-1) [64]. This is important because IGF-1 controls glucose and energy metabolism in the human body [65]. These reports have provided a rationale for the involvement of HOTAIR in glucose metabolism and insulin resistance through targeting different genes. Therefore, *HOTAIR* gene polymorphisms might be a predictor for other types of diabetes or related complications. However, to this day, not a single study has reported the link between functional gene variations in *HOTAIR* and other types of diabetes.

Lately, genetic association studies have gained much attention for discovering the genetic basis of complex metabolic disorders. In this regard, multiple genes have been linked with the risk of developing T2DM through genome-wide scanning, and advancement in identifying potentially causal variations for such disorders holds great promise. This suggesting that understanding the heterogeneity of diabetes provides an opportunity for personalized prevention and developing novel treatment strategies for cases with a certain monogenic form of the disease [66]. The present study's findings highlighted the role of *HOTAIR* variations on the risk of developing T2DM as an expanding global health challenge. However, replication studies with similar case-control designs on larger populations and diverse races are needed to confirm these results. Moreover, functional studies are warranted to explain the underlying mechanism of such associations.

The main SNPs in lncRNA sequences and their relation with cancer risk in several types of solid tumors have been demonstrated [67]. Additionally, the circulating lncRNAs are indicative of various diseases; some of these are unique, but several are common in several diseases and as novel diagnostic and prognostic biomarkers of human diseases [68]. Many recent studies have shown that lncRNAs participate in the occurrence and development of other human diseases and diabetes and play critical regulatory roles [69]. Research articles on the significance of *HOTAIR* have been widely published in the literature, primarily in cancer. But its polymorphism is a new approach in diabetes [70]. Different studies illustrated the association between *HOTAIR* SNPs and the genetic susceptibility to various cancers [71]. Aglan et al., in 2021, reported that the presence of *HOTAIR* rs12826786 C > T polymorphism could be used to assess the risk of females for developing breast cancer and might be of potential benefit in screening the disease [71]. The outcome of these reports have shed light on the clinical significance of HOTAIR, a newly found lncRNA, and necessity to detect genetic variations within this gene in different populations.

Briefly, aberrant expression of *HOTAIR* is frequently associated with pathogenesis and mostly with metastatic progression of several human cancers. Different polymorphisms, particularly present in intronic sequences and promoter regions of *HOTAIR*, are often associated with its aberrant expression, patient prognosis, and cancer susceptibility in different tumor phenotypes [67]. We hypothesize that this approach could be expanded in other disorders and diabetes. *HOTAIR* polymorphism can be detected in diabetic patients and considered as diagnostic, prognostic, and therapeutic biomarkers. Besides, HOTAIR and fast blood glucose were independent biomarkers of T2DM, respectively [69].

We used the PCR technique to identify subjects with *HOTAIR* polymorphisms our populations with greater than 99% accuracy. This can be applied to larger populations, and both ARMS-PCR and PCR-RFLP assays can be easily performed in most laboratories, results are available in a short period, and most importantly, the cost of performing such methods is much less than that of next-generation sequencing (NGS), HLA genotyping-based association analysis, or SNP arrays. Employing PCR-based techniques makes greatly reduces the cost and time needed to genotype a large group of samples to identify such genetic biomarkers associated with T2DM risk. However, compared with more advanced techniques such as NGS or pyrosequencing, genotyping methods employed in the current study has less genotyping accuracy, which can be considered a challenge that needs to be tackled. However, according to our hypothesis, *HOTAIR* polymorphism detection, within the routine diagnostic and prognostic tests, can be considered not only as complementary theragnostic biomarkers but also could be used as independent tests in “Personalized Medicine.” It means that each diabetic patient might be followed based on his/her *HOTAIR* SNP profile.

In conclusion, we firstly reported that *HOTAIR* rs1899663 G/T, rs12826786 C/T, rs4759314 A/G, and rs920778 C/T polymorphisms might influence T2DM susceptibility by modulating different signaling pathways and could be regarded as potential prognostic markers in T2DM patients.

## Figures and Tables

**Table 1:** Primers and methods used for genotyping of *HOTAIR* polymorphisms.

SNP	Genotyping method	Primer sequence	Annealing temperature	RE	Product size (bp)
rs1899663 G/T	ARMS-PCR	F (C-allele): AAAGCCTCTAATTGTTGTCATC F (A-allele): AAAGCCTCTAATTGTTGTCATA R: AGACCCTCAGGTCCCTAATA	55°C	—	C and A: 207
rs12826786 C/T	ARMS-PCR	F: AGACCTTGGTCCAATTCCCC R (G allele): AGAGGGAAGGAGCTTAGGATAAACG R (A-allele): AGAGGGAAGGAGCTTAGGATAAACA	62°C	—	G and A: 364
rs4759314 A/G	PCR-RFLP	F: TTCAGGTTTTATTAACTTGCATCAGC R: ACCCAAAACCATTTCCTGAGAG	55°C	*AluI*	G: 124 A: 99 + 25
rs920778 C/T	PCR-RFLP	F: TTACAGCTTAAATGTCTGAATGTTCC R: GCCTCTGGATCTGAGAAAGAAA	56°C	*MspI*	T: 140 C: 113 + 27

F: forward; R: reverse; SNP: single-nucleotide polymorphism; RFLP-PCR: restriction fragment length polymorphism polymerase chain reaction; ARMS-PCR: amplification refractory mutation system polymerase chain reaction; bp: base pair; RE: restriction enzyme.

**Table 2:** Clinical and demographic features of T2DM patients and healthy controls.

Parameter evaluated	T2DM (*n* = 500) (mean ± SD)	Controls (*n* = 500) (mean ± SD)	*p* value
Age (year)	55.28 ± 10.20	54.87 ± 11.15	0.066
Sex (female/male)	357/145	345/162	0.290
BMI (kg/m^2^)	32.55 ± 77.31	21.64 ± 2.26	<0.001
FBS (mg/dL)	182.36 ± 70.44	96.93 ± 19.25	<0.001
TG (mg/dL)	162.01 ± 84.93	151.27 ± 101.28	<0.001
TC (mg/dL)	179.16 ± 43.19	180.90 ± 37.43	0.181
HDL-C (mg/dL)	56.22 ± 19.17	54.10 ± 14.90	0.232
LDL-C (mg/dL)	96.37 ± 32.30	104.86 ± 28.73	<0.001
HbA1C (%)	9.16 ± 6.22	4.73 ± 0.76	<0.001

BMI: body mass index; FBS: fast blood sugar; TC: total cholesterol; TG: triglyceride; HDL-C: high-density lipoprotein-cholesterol; LDL-C: low-density lipoprotein-cholesterol; T2DM: type 2 diabetes; kg: kilogram; m^2^: square meter; mg: milligram; dL: deciliter. *p* < 0.05 was considered statistically significant.

**Table 3:** Allelic and genotypic distribution of *HOTAIR* polymorphisms.

SNP	T2DM, *n* (%)	Control, *n* (%)	Genetic model	OR (95% CI)	*p* value
rs920778 C/T					
CC	182 (36.3)	220 (43.4)		1 (reference)	
CT	245 (48.8)	240 (47.3)	Codominant 1	1.23 (0.95-1.61)	0.120
TT	75 (14.9)	47 (9.3)	Codominant 2	1.93 (1.27-2.92)	0.002
			Dominant	1.35 (1.05-1.74)	0.021
			Recessive	1.72 (1.17-2.53)	0.006
			Over dominant	1.06 (0.83-1.36)	0.641
C	609 (60.7)	680 (67.1)	Allelic	1 (reference)	
T	395 (39.3)	334 (32.9)	Allelic	1.32 (1.10-1.58)	0.003
rs1899663 G/T					
GG	202 (40.2)	184 (36.3)		1 (reference)	
GT	261 (52.0)	254 (50.1)	Codominant 1	0.94 (0.72-1.22)	0.623
TT	39 (7.8)	69 (13.6)	Codominant 2	0.51 (0.33-0.80)	0.003
			Dominant	0.85 (0.66-1.09)	0.197
			Recessive	0.53 (0.35-0.81)	0.003
			Over dominant	1.08 (0.84-1.38)	0.547
G	665 (66.2)	622 (61.3)	Allelic	1 (reference)	
T	339 (33.8)	392 (38.7)	Allelic	0.81 (0.67-0.97)	0.022
rs12826786 C/T					
CC	161 (32.1)	198 (39.1)		1 (reference)	
CT	251 (50.0)	247 (48.7)	Codominant 1	1.25 (0.95-1.64)	0.109
TT	90 (17.9)	62 (12.2)	Codominant 2	1.79 (1.21-2.62)	0.003
			Dominant	1.36 (1.05-1.76)	0.020
			Recessive	1.57 (1.10-2.22)	0.010
			Over dominant	1.05 (0.82-1.35)	0.680
C	573 (57.1)	643 (63.4)	Allelic	1 (reference)	
T	431 (42.9)	371 (36.3)	Allelic	1.30 (1.09-1.56)	0.003
rs4759314 A/G					
AA	191 (38.0)	215 (42.4)		1 (reference)	
AG	238 (47.4)	241 (57.5)	Codominant 1	1.11 (0.85-1.45)	0.433
GG	73 (14.5)	51 (10.1)	Codominant 2	1.61 (1.07-2.42)	0.021
			Dominant	1.20 (0.93-1.54)	0.158
			Recessive	1.52 (1.04-2.23)	0.030
			Over dominant	0.99 (0.78-1.27)	0.969
A	620 (61.8)	671 (66.2)	Allelic	1 (reference)	
G	384 (38.2)	343 (33.8)	Allelic	1.21 (1.01-1.45)	0.038

T2DM: type 2 diabetes mellitus; SNP: single-nucleotide polymorphism; CI: confidence interval; OR: odds ratio. Codominant 1 and codominant 2 represent the heterozygous and homozygous codominant models, respectively. *p* < 0.05 is considered statistically significant.

**Table 4:** Association between *HOTAIR* cluster variants and clinical-demographic characteristics of T2DM patients and healthy controls.

Variable	Genotype	FBS (mg/dL)	TC (mg/dL)	TG (mg/dL)	HDL-C (mg/dL)	LDL-C (mg/dL)
rs920778 C/T						
T2DM	TT TC + CC *p* value	186.48 ± 77.38 181.64 ± 69.23 0.763	184.50 ± 42.95 184.99 ± 48.26 0.877	180.11 ± 98.78 158.83 ± 81.97 0.108	52.21 ± 21.41 56.92 ± 18.69 *0.016*	102.33 ± 36.88 95.31 ± 31.35 0.161
Control	TT TC + CC *p* value	98.90 ± 15.03 96.75 ± 19.58 0.139	181.12 ± 33.09 181.47 ± 37.71 0.655	135.57 ± 56.56 130.94 ± 73.78 0.305	48.89 ± 16.54 47.11 ± 17.68 0.886	100.89 ± 25.71 105.17 ± 28.96 0.589
rs1899663 G/T						
T2DM	TT TG + GG *p* value	170.05 ± 65.81 183.40 ± 70.79 0.195	178.95 ± 35.79 185.42 ± 48.31 0.617	163.41 ± 85.91 161.90 ± 84.94 0.940	59.82 ± 19.66 55.93 ± 19.12 0.207	91.38 ± 20.68 96.79 ± 33.09 0.585
Control	TT TG + GG *p* value	94.17 ± 14.73 97.32 ± 19.79 0.058	175.03 ± 35.81 182.39 ± 37.49 0.127	132.45 ± 60.43 131.17 ± 74.12 0.514	50.27 ± 14.58 46.89 ± 17.88 0.243	101.44 ± 30.40 105.37 ± 28.48 0.520
rs12826786 C/T						
T2DM	TT CT + CC *p* value	184.87 ± 72.68 181.82 ± 70.02 0.773	181.48 ± 44.71 185.67 ± 48.06 0.585	149.92 ± 66.73 164.66 ± 88.25 0.451	60.05 ± 22.87 55.37 ± 18.17 0.120	94.45 ± 32.38 96.79 ± 32.31 0.730
Control	TT CT + CC *p* value	95.45 ± 12.71 97.14 ± 20.00 0.780	179.62 ± 30.70 181.70 ± 38.18 0.662	129.49 ± 58.42 131.60 ± 74.35 0.971	57.10 ± 13.50 46.16 ± 17.61 *<0.001*	96.33 ± 30.68 106.11 ± 28.25 0.065
rs4759314 A/G						
T2DM	GG AA + AG *p* value	200.27 ± 71.73 197.32 ± 69.85 *0.008*	183.20 ± 46.42 185.21 ± 47.68 0.806	174.70 ± 98.02 159.86 ± 82.43 0.403	53.92 ± 17.85 56.61 ± 19.38 0.466	89.72 ± 27.70 97.52 ± 32.92 *0.043*
Control	GG AA + AG *p* value	95.63 ± 13.44 97.04 ± 19.69 0.602	168.88 ± 32.27 182.57 ± 37.57 *0.041*	106.27 ± 55.35 133.62 ± 73.43 *0.004*	48.61 ± 17.59 47.13 ± 17.59 0.481	102.36 ± 21.25 105.11 ± 29.37 0.573

FBS: fast blood sugar; TC: total cholesterol; TG: triglyceride; HDL-C: high density lipoprotein-cholesterol; LDL-C: low density lipoprotein-cholesterol; T2DM: type 2 diabetes mellitus. *p* values were adjusted for sex, age, and BMI. *p* < 0.05 was considered statistically significant (italicized *p* value).

**Table 5:** Haplotype analysis of *HOTAIR* SNPs between T2DM patients and controls.

rs920778 C/T	rs1899663 G/T	rs12826786 C/T	rs4759314 A/G	T2DM, *n* (%)	Control, *n* (%)	*p* value (OR (95% CI))
C	G	C	A	170 (17.0)	175 (17.3)	1 (reference)
C	G	C	G	98 (9.8)	65 (6.5)	0.022 (1.55 (1.06-2.27))
C	G	T	A	98 (9.8)	89 (8.8)	0.490 (1.13 (0.79-1.62))
C	G	T	G	54 (5.3)	80 (7.9)	0.077 (0.69 (0.46-1.04))
C	T	C	A	51 (5.1)	113 (11.2)	<0.001 (0.46 (0.31-0.69))
C	T	C	G	51 (5.1)	61 (6.1)	0.491 (0.86 (0.56-1.32))
C	T	T	A	48 (4.8)	75 (7.4)	0.050 (0.66 (0.43-1.00))
C	T	T	G	38 (3.8)	20 (2.0)	0.022 (1.96 (1.09-3.50))
T	G	C	A	80 (7.9)	109 (10.7)	0.124 (0.76 (0.53-1.08))
T	G	C	G	44 (4.4)	39 (3.8)	0.541 (1.16 (0.72-1.88))
T	G	T	A	82 (8.2)	30 (3.0)	<0.001 (2.81 (1.76-4.49))
T	G	T	G	38 (3.8)	34 (3.4)	0.589 (1.15 (0.69-1.91))
T	T	C	A	45 (4.5)	43 (4.3)	0.755 (1.08 (0.67-1.72))
T	T	C	G	34 (3.3)	37 (3.6)	0.831 (0.95 (0.57-1.58))
T	T	T	A	45 (4.4)	36 (3.5)	0.309 (1.29 (0.79-2.09))
T	T	T	G	28 (2.7)	6 (0.6)	<0.001 (4.80 (1.94-11.89))

T2DM: type 2 diabetes mellitus; CI: confidence interval; OR: odds ratio. *p* < 0.05 is considered statistically significant.

**Table 6:** Analysis of linkage disequilibrium between *HOTAIR* polymorphisms (case and control).

SNP	*D*′ statistics			*r* statistic		
	rs1899663	rs12826786	rs4759314	rs1899663	rs12826786	rs4759314
rs920778	0.018	0.032	0.008	0.000	0.001	0.000
rs1899663	—	0.008	0.015	—	0.000	0.000
rs12826786	—	—	0.012	—	—	0.000

SNP: single nucleotide polymorphism.

## Data Availability

The data in this manuscript are available from the corresponding author upon reasonable request.
